# Treatment with paeoniflorin increases lifespan of *Pseudomonas aeruginosa* infected *Caenorhabditis elegans* by inhibiting bacterial accumulation in intestinal lumen and biofilm formation

**DOI:** 10.3389/fphar.2023.1114219

**Published:** 2023-03-27

**Authors:** Yuxing Wang, Le Zhang, Xiaoan Yuan, Dayong Wang

**Affiliations:** Jiangsu Provincial Key Laboratory of Critical Care Medicine, Medical School, Southeast University, Nanjing, China

**Keywords:** *P. aeruginosa*, paeoniflorin, colony, biofilm, *C. elegans*

## Abstract

Paeoniflorin is one of the important components in *Paeoniaceae* plants. In this study, we used *Caenorhabditis elegans* as a model host and *Pseudomonas aeruginosa* as a bacterial pathogen to investigate the possible role of paeoniflorin treatment against *P. aeruginosa* infection in the host and the underlying mechanisms. Posttreatment with 1.25–10 mg/L paeoniflorin could significantly increase the lifespan of *P. aeruginosa* infected nematodes. After the infection, the *P. aeruginosa* colony-forming unit (CFU) and *P. aeruginosa* accumulation in intestinal lumen were also obviously reduced by 1.25–10 mg/L paeoniflorin treatment. The beneficial effects of paeoniflorin treatment in increasing lifespan in *P. aeruginosa* infected nematodes and in reducing *P. aeruginosa* accumulation in intestinal lumen could be inhibited by RNAi of *pmk-1*, *egl-1*, and *bar-1*. In addition, paeoniflorin treatment suppressed the inhibition in expressions of *pmk-1*, *egl-1*, and *bar-1* caused by *P. aeruginosa* infection in nematodes, suggesting that paeoniflorin could increase lifespan of *P. aeruginosa* infected nematode by activating PMK-1, EGL-1, and BAR-1. Moreover, although treatment with 1.25–10 mg/L paeoniflorin did not show obvious anti-*P. aeruginosa* activity, the *P. aeruginosa* biofilm formation and expressions of related virulence genes (*pelA*, *pelB*, *phzA*, *lasB*, *lasR*, *rhlA*, and *rhlC*) were significantly inhibited by paeoniflorin treatment. Treatment with 1.25–10 mg/L paeoniflorin could further decrease the levels of related virulence factors of pyocyanin, elastase, and rhamnolipid. In addition, 2.5–10 mg/L paeoniflorin treatment could inhibit the swimming, swarming, and twitching motility of *P. aeruginosa*, and treatment with 2.5–10 mg/L paeoniflorin reduced the cyclic-di-GMP (c-di-GMP) level. Therefore, paeoniflorin treatment has the potential to extend lifespan of *P. aeruginosa* infected hosts by reducing bacterial accumulation in intestinal lumen and inhibiting bacterial biofilm formation.

## Introduction

*Pseudomonas aeruginosa* is a Gram-negative opportunistic bacterial pathogen. *P. aeruginosa* infection is associated with some diseases, such as sepsis, by causing severe health problems (Cheluvappa et al., 2009; Berube et al., 2016). *P. aeruginosa* has extraordinary capacity to infect multiple organs and tissues, such as intestinal infection and lung infection, which suggests the potential induction of systemic infections by *P. aeruginosa* (Markou and Apidianakis, 2014; Curran et al., 2018). In addition, *P. aeruginosa* infection also affect blood circulation and circulating regulatory T-cells (Westhölter et al., 2021). *P. aeruginosa* infection can further alter functions of immune cells and their immune response (Alhazmi, 2018). So far, the complex mechanisms for *P. aeruginosa* infection and immune response of host have been extensively investigated (Malhotra et al., 2019; Moser et al., 2021).

*Caenorhabditis elegans* is a classic model animal with short life-cycle and lifespan, and sensitive to various environmental exposures (Wang, 2020; Xu et al., 2022b; Zhao Y.-Y. et al., 2023; Zhao Y.-L. et al., 2023). The powerful genetic platform makes *C. elegans* convenient to tract behavior of pathogen infection in their gut (Balla and Troemel, 2013; Kim and Ewbank, 2018). In their natural habitat, *C. elegans* can encounter various microbes, including both bacterial and fungal pathogens (Kim and Ausubel, 2005). Thus, *C. elegans* is a useful animal model for the study of host-pathogen interactions. In *C. elegans*, after the infection, innate immunity is normally activated in primary biological barriers (epidermis and the intestine) against toxic effects of pathogens (Taffoni and Pujol, 2015), which allows the nematodes reproduce successfully and survive long enough (Martineau et al., 2021). In *C. elegans*, the activated innate immune response is reflected by the secreted antimicrobial proteins to kill the pathogens (Engelmann and Pujol, 2010). Meanwhile, some molecular signaling pathways (such as p38 MAPK, insulin, Wnt, and TGF-β) will also be activated to modulate the pathogen infection (Kurz and Tan, 2004; Irazoqui et al., 2008; Arvanitis et al., 2013; Head et al., 2017; Yu et al., 2018).

Due to the easy of cultivation and automation of transfer and image acquisition, *C. elegans* has been frequently used for high throughput drug screen (Kwok et al., 2006; Lehner et al., 2006). Meanwhile, the well-described genetic and molecular backgrounds make *C. elegans* suitable for elucidating pharmacological mechanism of certain bioactive compounds (Griffin et al., 2017; Okoro et al., 2021). *C. elegans* can be further used as a host to perform whole-organism screen to identify novel compounds having antivirulent property (Kirienko et al., 2013). For example, based on high-content screen together with phenotypic analysis in *C. elegans*, 5-fluorouracil and its downstream metabolite 5-fluorouridine were identified as antivirulent compounds (Kirienko et al., 2016).

Paeoniflorin belongs to monoterpenoid glycoside, which was initially extracted from *Paeonia lactiflora* Pall (Zhang et al., 2019). In other *Paeoniaceae* plants, the paeoniflorin can also be detected. Previous studies have shown some aspects of beneficial effects from paeoniflorin treatment, including neuroprotection, anti-tumor, anti-oxidation, and anti-depression (Li et al., 2017; Zhong et al., 2018; Ma et al., 2020). Besides these, the paeoniflorin treatment has been further shown to show anti-inflammatory function (Zhang and Wei, 2020). We assumed that paeoniflorin treatment may have the beneficial effect against bacterial infection in the hosts. In this study, *C. elegans* was used as a genetic host, and *P. aeruginosa* was selected as a pathogen. Using this host and the bacterial pathogen, we examined whether the paeoniflorin treatment has the beneficial effect of anti-bacterial infection. Moreover, the underlying mechanism for this possible anti-infection function of paeoniflorin was determined. Our results demonstrate the beneficial effect of paeoniflorin treatment against *P. aeruginosa* infection by suppressing both colony accumulation in intestinal lumen and bacterial biofilm formation. Our data suggested the potential of paeoniflorin used for inhibiting *P. aeruginosa* infection in hosts.

## Materials and methods

### *C. elegans* maintenance

The strain used in this study was wild-type N2, which was maintained on nematode growth medium (NGM) plates fed with *Escherichia coli* OP50 as the food source (Brenner, 1974). To obtain enough young adults for bacterial infection, we treated the pregnant hermaphroditic nematodes with lysis solution containing 0.45 M NaOH and 2% HOCl to release eggs from the body (Zhao Y.-Y. et al., 2022). After that, the eggs were collected and transferred onto the surface of new NGM plate to allow them develop into synchronized young adults.

### *P. aeruginosa* preparation and infection

The used *P. aeruginosa* strains were PA14 and PA14:GFP. PA14 is a normally used *P. aeruginosa* strain for the study of innate immunity in *C. elegans* (Zhi et al., 2017a). These strains were cultured in Luria broth. The full lawn of PA14 or PA14:GFP killing plates were prepared by seeding them on modified NGM killing plates containing 0.35% peptone. After the seeding, the killing plates were incubated for at 37°C for 24-h and further at 25°C for 24-h. Young adults were transferred on killing plate to perform the *P. aeruginosa* PA14 infection at 25°C for 24-h (Zhi et al., 2017b). Three replicates were performed.

### Pharmacological treatment

**FIGURE 1:**
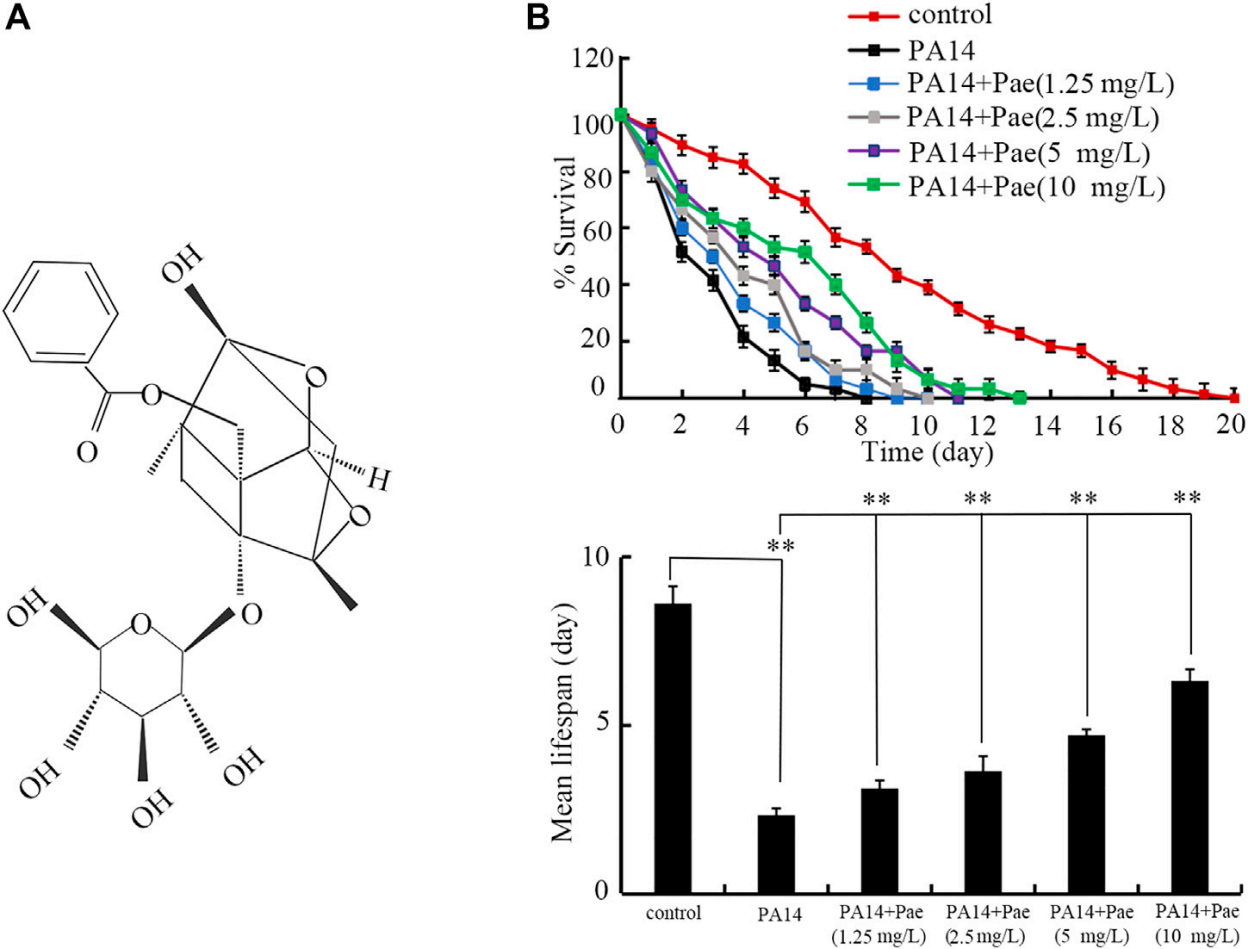
Effect of paeoniflorin treatment in increasing lifespan in *P. aeruginosa* PA14 infected nematodes. **(A)** Chemical structure of paeoniflorin. **(B)** Effect of paeoniflorin treatment on lifespan of nematodes infected with *P. aeruginosa* PA14. Lifespan curve of PA14 showed a significant difference (*p* < 0.01) compared to control. Lifespan curves of PA14 + Pae (1.25 mg/L), PA14 + Pae (2.5 mg/L), PA14 + Pae (5 mg/L), and PA14 + Pae (10 mg/L) showed a significant difference (*p* < 0.01) compared to PA14. Pae, paeoniflorin. **p < 0.01.

Chemical structure of paeoniflorin is shown in Figure 1A. After *P. aeruginosa* PA14 infection, the nematodes were transferred into paeoniflorin to perform the posttreatment for 24-h. The paeoniflorin treatment concentrations were 1.25, 2.5, 5, and 10 mg/L. The concentration selection was largely based on previously published report (Zhang et al., 2018). Three replicates were performed.

### Lifespan assay

To test the effect of *P. aeruginosa* PA14 infection and paeoniflorin posttreatment on lifespan, the number of dead nematodes was counted. The examined nematodes were counted as dead if no response was detected after prodding using platinum wire. The mean lifespan is defined as the day at which 50% animals survived. Fifty animals were tested for each group. Statistical significance between survival curves was examined by Kaplan-Meier survival analysis, followed by the log-rank test.

### Assay of reactive oxygen species (ROS) production and locomotion behavior

ROS production was used to reflect the activation of oxidative stress in nematodes (Zhang et al., 2022b). To analyze intestinal ROS production, the nematodes were labeled with 1 μM CM-H_2_DCFDA for 3 h in the dark (Hua et al., 2023a). After the labelling, the nematodes were washed with M9 buffer to remove excess probes. Fluorescent signals were detected using a laser confocal microscope at 488/510 nm (excitation/emission wavelengths). ROS production was assessed by intestinal fluorescence intensity after normalization to the intestinal autofluorescence. Fifty animals were analyzed for each treatment.

Locomotion behaviors, head thrash and body bend, were used to reflect the function of motor neurons in nematodes (Zhang et al., 2022c). A body bending was counted as the change of direction for bending at the mid-body (Xu et al., 2022a). A head thrashing was counted as the change of direction for the posterior bulb (y-axis), if we considered the direction of swimming of the nematodes as the x-axis (Liu et al., 2022). Fifty animals were analyzed for each treatment.

### Assay of colony-forming unit (CFU)

To examine the CFU of *P. aeruginosa* PA14 in the body of nematodes, the adult nematodes after the infection were first treated with 25 mM levamisole in order to block pharyngeal pumping of animals. To eliminate *P. aeruginosa* PA14 on the body surface, the nematodes were further transferred onto NGM plate containing gentamicin (1 mg/mL) and ampicillin (1 mg/mL) to treat for 30-min. After these treatments, fifty nematodes for each group were lysed using motorized pestle and transferred on Luria-Bertani (LB) plates containing rifampicin (100 μg/mL) to incubate at 37°C overnight. The colony number of *P. aeruginosa* PA14 was counted for each plate. Five replicates were performed.

To confirm the accumulation of *P. aeruginosa* PA14 in intestinal lumen, we also used the *P. aeruginosa* strain of PA14:GFP to directly visualize *P. aeruginosa* PA14 accumulation in nematodes (Zhang et al., 2022a). The data was expressed as relative fluorescence intensity of *P. aeruginosa* PA14:GFP in intestinal lumen after normalization to intestinal autofluorescence of nematodes. Fifty animals were examined for each group, and three replicates were performed.

### Assay of quantitative real-time polymerase chain reaction (qRT-PCR)

The reagent TRIzol was used to extract the total RNA of both adult nematodes and *P. aeruginosa* PA14. In NanoDrop One, quality of extracted *C. elegans* and *P. aeruginosa* RNAs were assessed by OD260/280 ratio. The *C. elegans* and *P. aeruginosa* cDNAs were synthesized in a gradient MasterCycler (Eppendorf, United States). The qRT-PCR was carried out using SYBR Green Master Mix in an ABI 7500 real-time PCR system. The method of comparative CT (ΔΔ CT) was used to analyze alterations in transcriptional expressions of examined genes after the normalization with expression of reference gene (*tba-1* for *C. elegans* and *pvdQ* for *P. aeruginosa*) (Yang D. et al., 2021; Zhao Y. et al., 2022). Three replicates were performed. The information of designed primers is given in Supplementary Tables S1, S2.

### RNA interference (RNAi)

To knockdown expression of certain gene(s), RNAi experiments were performed (Yang Y.-H. et al., 2021). RNAi constructs were generated using empty vector L4440, and transformed into *E. coli.* HT115. The RNAi was carried out by feeding the nematodes with *E. coli* HT115 expressing a certain gene after *P. aeruginosa* PA14 infection. *E. coli* HT115 expressing empty vector L4440 was used as the control (Hua et al., 2022). Three independent experiments were performed. The RNAi efficiency for RNAi of *daf-16*, *dbl-1*, *elt-2*, *pmk-1*, *egl-1*, and *bar-1* was reported previously (Zhang et al., 2022a).

### Analysis of antibacterial activity

1) Time-kill assay. After culturation overnight, *P. aeruginosa* PA14 was centrifuged and then dispersed into 1.25–10 mg/L paeoniflorin in a volume of 5 mL. The *P. aeruginosa* PA14 was further incubated at 35°C. The colony number was counted at 0, 6, 12, 18, and 24 h. The 1 μg/mL ampicillin was used as the positive control. The experiments were repeated for three times.
2) Agar diffusion assay. After culturation overnight, *P. aeruginosa* PA14 was centrifuged and washed with PBS buffer. In liquid LB medium, approximately 10^7^ *P. aeruginosa* PA14 cells/mL were inoculated. The suspensions with the volume of 10 mL were transferred on the LB agar plate. The paeoniflorin solutions (1.25–10 mg/L) were added onto filter disks (diameter, 6-mm), which were placed on the agar surface to incubate at 35°C for 48 h. The 1 μg/mL ampicillin was used as the positive control. The experiments were repeated for three times.

### *P. aeruginosa* PA14 biofilm formation

The biofilm formation of *P. aeruginosa* PA14 was firstly analyzed by crystal violet method (Lee et al., 2011). The *P. aeruginosa* cells (approximately 5 × 10^5^ CFU/mL) were transferred in a 96-well plate to incubate together with 1.25–10 mg/L paeoniflorin at 37°C for 36-h. After the washing with PBS buffer, the *P. aeruginosa* biofilm was fixed with methanol for 15-min. The *P. aeruginosa* biofilm was then stained by crystal violet for 15-min. After the staining, the biofilm was dried at 60°C for 1 h, and dissolved with 200 μL acetic acid (33%) for 15-min. The absorbance was also analyzed to quantify *P. aeruginosa* biofilm formation at 595 nm. In addition, the *P. aeruginosa* biofilm formation was further directly visualized under a light microscope. The experiments were repeated for three times.

### Pyocyanin assay

The pyocyanin production in *P. aeruginosa* was analyzed as described (Chong et al., 2011). Totally 2 mL of *P. aeruginosa* PA14 cells (approximately 5 × 10^5^ CFU/mL) were incubated with 1.25–10 mg/L paeoniflorin at 37°C for 48-h. The *P. aeruginosa* PA14 suspensions were centrifugated, and the supernatants were extracted using 0.75 mL chloroform. The 0.25 mL HCl (0.2 M) was added on the chloroform layer. The mixture was further centrifugated and the HCl was removed. The absorbance at 520 nm was determined for pyocyanin quantification. The experiments were repeated for three times.

### Elastase activity

The elastase activity of *P. aeruginosa* PA14 was determined as described (Adonizio et al., 2008). Briefly, 2 mL of *P. aeruginosa* PA14 cells (approximately 5 × 10^5^ CFU/mL) were incubated with 1.25–10 mg/L paeoniflorin at 37°C for 24-h. The *P. aeruginosa* PA14 suspensions were centrifugated at 12,000 rpm for 10 min, and filter-purified by a 0.22 µm nylon filter. After that, the supernatant (100 µL) was added together with 20 mg ECR dissolved in 400 µL ECR buffer (100 mM Tris, 1 mM calcium chloride, pH 7.2) to incubate at 37°C fir 16-h. The absorbance at 495 nm for the supernatant was determined. The experiments were repeated for three times.

### Rhamnolipid assay

The rhamnolipids in *P. aeruginosa* was analyzed as described (Yang D. et al., 2021). Totally 2 mL of *P. aeruginosa* PA14 cells (approximately 5 × 10^5^ CFU/mL) were incubated with 1.25–10 mg/L paeoniflorin at 37°C for 24-h. After that, the *P. aeruginosa* PA14 suspensions were centrifugated, and the supernatants were extracted twice using ethyl acetate. The organic layer was collected and evaporated overnight at 50°C. The solid products were dissolved in sterile distilled water (500 µL), and 100 µL of them was added with 0.19% ice alcohol in 53% concentrated sulfuric acid to incubate at 80°C for 30-min. After the cooling, absorbance at 421 nm was determined for the examined samples. The experiments were repeated for three times.

### *P. aeruginosa* PA14 motility assay

The method for swimming motility was performed as described (Ha et al., 2014). In *P. aeruginosa*, the swimming motility is defined as the movement in low-viscosity conditions (up to 0.3% agar concentration). The sterile toothpick was dipped in the overnight culture and stabbed into the center of the agar layer of the plates. The plates were incubated upright at 37°C for 24 h. The experiments were repeated for three times.

The method for swarming motility was performed as described (Zahmatkesh et al., 2022). Swimming agar plates containing 0.3% agar with the addition of paeoniflorin in Luria Broth was prepared. A sterile toothpick was dipped in the overnight culture and stabbed into the center of plates. After incubation of the plates at 37°C for 24 h, the swimming motility was assessed. The experiments were repeated for three times.

The method for twitching motility was performed as described (Zahmatkesh et al., 2022). Twitching agar plates containing 1.5% agar with the addition of paeoniflorin in Luria Broth were prepared. The match-head-sized colonies from the overnight culture were inoculated to the bottom of the plates. After incubation of the plates at 37°C for 24 h, the twitching motility zone was assessed. The experiments were repeated for three times.

### Data analysis

Data are presented as means ± SD. SPSS 12.0 software was used for statistical analysis. Differences between different groups were analyzed by analysis of variance (ANOVA). A probability level of 0.01 was considered statistically significant.

## Results

### Effect of paeoniflorin treatment in increasing lifespan of *P. aeruginosa* PA14 infected nematodes

Using lifespan as the endpoint, we investigated the possible beneficial effect of paeoniflorin posttreatment against *P. aeruginosa* PA14 infection in *C. elegans*. After the posttreatment, 1.25–10 mg/L paeoniflorin could significantly increase the lifespan of nematodes after *P. aeruginosa* PA14 infection (Figure 1B). The beneficial effect of paeoniflorin posttreatment in increasing lifespan of nematodes infected with *P. aeruginosa* PA14 was concentration dependent (Figure 1B).

Besides the endpoint of lifespan, we also used ROS production and locomotion behavior as endpoints to assess the pharmacological effect of paeoniflorin against *P. aeruginosa* PA14 infection in *C. elegans*. Infection with *P. aeruginosa* PA14 caused the obvious induction of ROS production and decrease in body bend frequency and head thrash frequency (Supplementary Figures S1A, B). Moreover, treatment with 2.5–10 mg/L paeoniflorin could significantly suppress the ROS production and increase the locomotion behavior reflected by body bend and head thrash in nematodes after *P. aeruginosa* PA14 infection (Supplementary Figures S1A, B).

### Effect of paeoniflorin treatment on bacterial colony formation in intestinal lumen of nematodes after the infection

**FIGURE 2:**
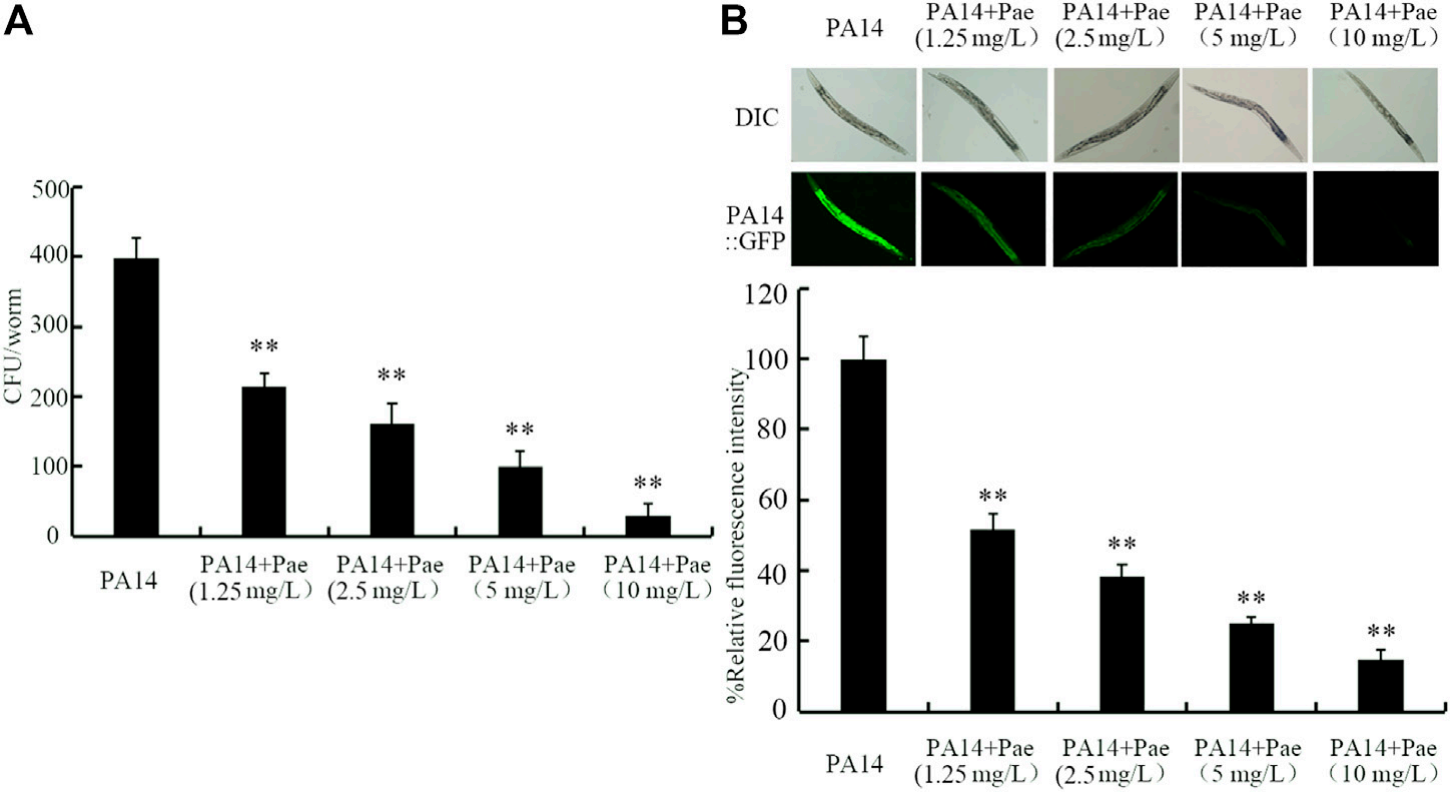
Effect of paeoniflorin treatment on CFU of *P. aeruginosa* PA14 **(A)** and accumulatio of *P. aeruginosa* PA14:GFP **(B)** in nematodes after the infection. Pae, paeoniflorin. **p < 0.01 vs*.* PA14.

To determine the underlying mechanisms for the observed beneficial effect of paeoniflorin treatment against *P. aeruginosa* PA14 infection, we first investigated the colony formation of PA14 in intestine. Treatment with paeoniflorin (1.25–10 mg/L) could obviously inhibit the formation of high intestinal CFU of *P. aeruginosa* PA14 in nematodes (Figure 2A). In addition, 1.25–10 mg/L paeoniflorin treatment could further significantly inhibit the *P. aeruginosa* PA14:GFP accumulation in intestinal lumen (Figure 2B). The beneficial effect of paeoniflorin treatment against the *P. aeruginosa* PA14 colony formation in intestinal lumen of nematodes was also concentration dependent (Figures 2A, B).

### Requirement of PMK-1, EGL-1, and BAR-1 for beneficial effect of paeoniflorin in increasing lifespan in nematodes after bacterial infection

**FIGURE 3:**
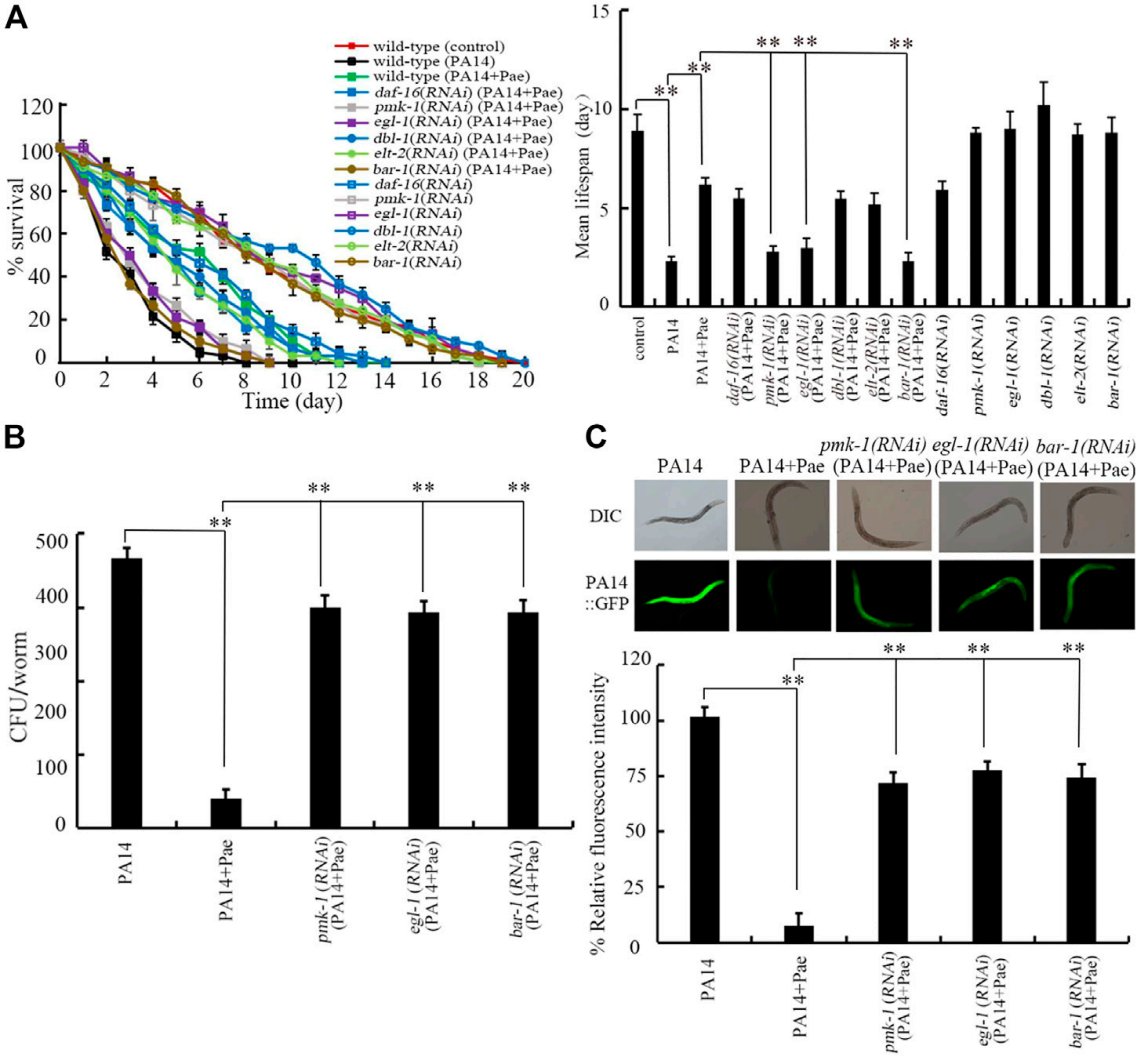
Effect of *bar-1*, *elt-2*, *pmk-1*, *dbl-1*, *egl-1*, and *daf-16* RNAi on beneficial role of 10 mg/L paeoniflorin in nematodes after the bacterial infection. **(A)** Effect of *bar-1*, *elt-2*, *pmk-1*, *dbl-1*, *egl-1*, and *daf-16* RNAi on beneficial role of paeoniflorin in increasing lifespan in nematodes after the bacterial infection. Lifespan curve of PA14 showed a significant difference (*p* < 0.01) compared to control. Lifespan curve of PA14 + Pae showed a significant difference (*p* < 0.01) compared to PA14. Lifespan curves of *pmk-1*(*RNAi*) (PA14 + Pae), *egl-1*(*RNAi*) (PA14 + Pae), and *bar-1*(*RNAi*) (PA14 + Pae) showed a significant difference (*p* < 0.01) compared to PA14 + Pae. In contrast, the lifespan curves of *daf-16*(*RNAi*) (PA14 + Pae) (*p* = 0.361), *dbl-1*(*RNAi*) (PA14 + Pae) (*p* = 0.364), and *elt-2*(*RNAi*) (PA14 + Pae) (*p* = 0.332) did not show a significant difference compared to PA14 + Pae. **(B)** Effect of *bar-1*, *elt-2*, *pmk-1*, *dbl-1*, *egl-1*, and *daf-16* RNAi on beneficial role of paeoniflorin in decreasing CFU of *P. aeruginosa* PA14 in nematodes after the bacterial infection. **(C)** Effect of *bar-1*, *elt-2*, *pmk-1*, *dbl-1*, *egl-1*, and *daf-16* RNAi on beneficial role of paeoniflorin in suppressing accumulatio of *P. =aeruginosa* PA14:GFP in nematodes after the bacterial infection. Pae, paeoniflorin. **p < 0.01.

Some molecular signals including insulin, Wnt, ELT-2, TGF-β, p38 MAPK, and PCD related signals have been identified to be involved in regulating the bacterial infection (Kurz and Tan, 2004; Irazoqui et al., 2008; Arvanitis et al., 2013; Zhi et al., 2017a; Head et al., 2017). We next investigated the possible involvement of these molecular signals in regulating pharmacological effect of paeoniflorin in increasing lifespan of *P. aeruginosa* PA14 infected nematodes. After *P. aeruginosa* PA14 infection, RNAi of *daf-16*, *dbl-1*, and *elt-2* did not affect the effect of paeoniflorin (10 mg/L) in increasing the lifespan in nematodes (Figure 3A). Different from this, after *P. aeruginosa* PA14 infection, RNAi of *pmk-1*, *egl-1*, and *bar-1* significantly inhibited the effect of paeoniflorin (10 mg/L) in increasing the lifespan of nematodes (Figure 3A). Therefore, PMK-1, EGL-1, and BAR-1 were required for the beneficial effect of paeoniflorin in extending lifespan in nematodes after *P. aeruginosa* PA14 infection. In *C. elegans*, DAF-16 in insulin signaling pathway, BAR-1 in Wnt signaling pathway, and ELT-2 are transcriptional factors, DBL-1 is a TGF-β ligand, PMK-1 is a p38 MAPK, and EGL-1 is a BH3 protein in PCD signaling pathway.

### Requirement of PMK-1, EGL-1, and BAR-1 for beneficial effect of paeoniflorin in reducing *P. aeruginosa* accumulation in intestinal lumen of nematodes

In addition, we also investigated the possible involvement of PMK-1, EGL-1, and BAR-1 in regulating pharmacological effect of paeoniflorin in reducing *P. aeruginosa* PA14 accumulation in nematodes. After the infection, RNAi of *pmk-1*, *egl-1*, and *bar-1* obviously suppressed the beneficial effect of paeoniflorin (10 mg/L) in reducing CFU in nematodes (Figure 3B). Similarly, after the infection, RNAi of *pmk-1*, *egl-1*, and *bar-1* noticeably inhibited the beneficial effect of paeoniflorin (10 mg/L) in suppressing PA14:GFP in intestinal lumen of nematodes (Figure 3B). Therefore, PMK-1, EGL-1, and BAR-1 were also required for the beneficial effect of paeoniflorin in reducing *P. aeruginosa* PA14 accumulation in intestinal lumen of nematodes.

### Effect of paeoniflorin treatment on expressions of *pmk-1*, *egl-1*, and *bar-1* in nematodes infected with *P. aeruginosa* PA14

**FIGURE 4:**
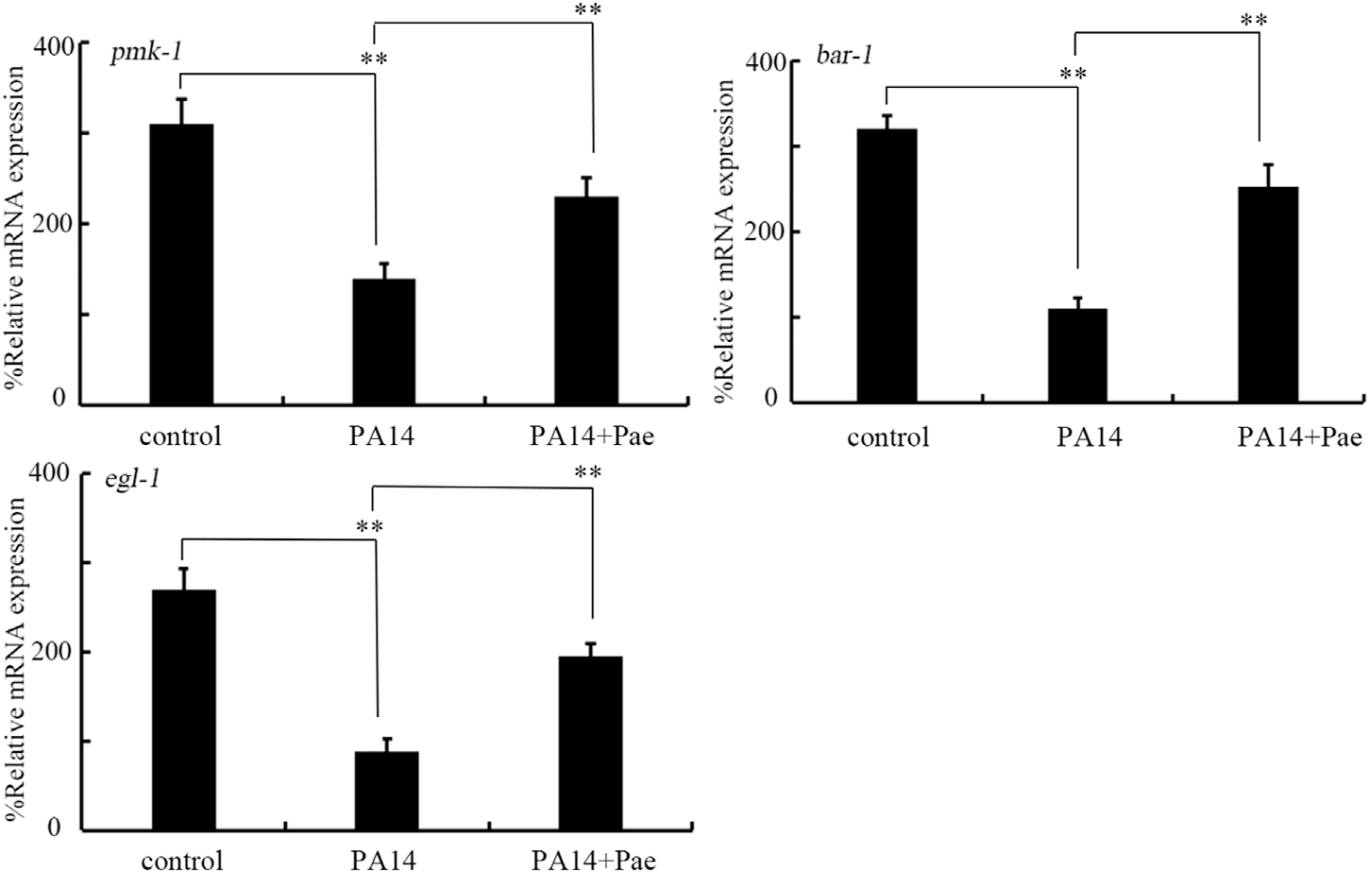
Effect of treatment with 10 mg/L paeoniflorin on expressions of *bar-1*, *pmk-1*, and *egl-1* in nematodes after *P. aeruginosa* PA14 infection. Pae, paeoniflorin. **p < 0.01.

In *C. elegans*, infection with *P. aeruginosa* PA14 significantly decreased the expressions of *pmk-1*, *egl-1*, and *bar-1* (Figure 4). Moreover, we observed that this inhibition in *pmk-1*, *egl-1*, and *bar-1* expressions by *P. aeruginosa* PA14 infection could be obviously suppressed by treatment with 10 mg/L paeoniflorin (Figure 4).

### Paeoniflorin treatment did not show obvious antibacterial activity

**FIGURE 5:**
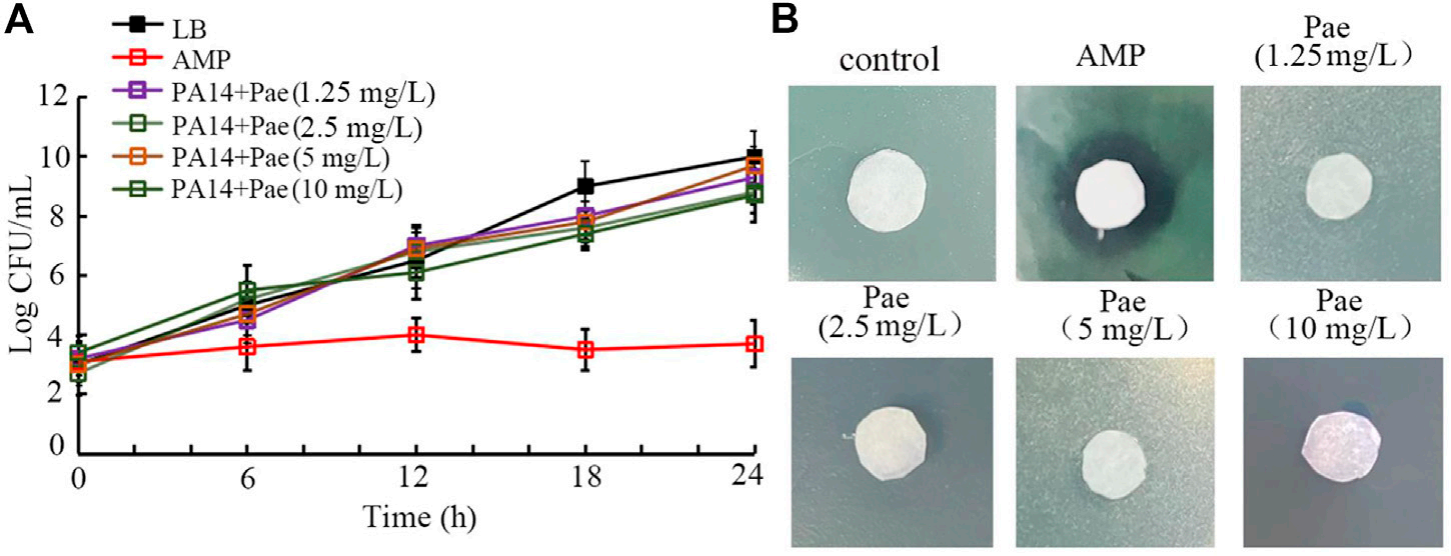
Analysis of the antibacterial activity of paeoniflorin. **(A)** Time-kill assay. **(B)** Disk diffusion assay. Pae, paeoniflorin. AMP, ampicillin. AMP treatment concentration was 1 μg/mL.

In the time-kill assay, treatment with 1.25–10 mg/L paeoniflorin all did not exhibit noticeable anti-*P. aeruginosa* PA14 activity at 6–24 h, which was very different from the control of 1 μg/mL ampicillin with strong anti-bacterial activity (Figure 5A). Moreover, in the agar diffusion assay, treatment with 1.25–10 mg/L paeoniflorin also could not cause the formation of obvious zone of inhibition as caused by 1 μg/mL ampicillin (Figure 5B).

### Paeoniflorin treatment inhibited biofilm formation of *P. aeruginosa* PA14

**FIGURE 6:**
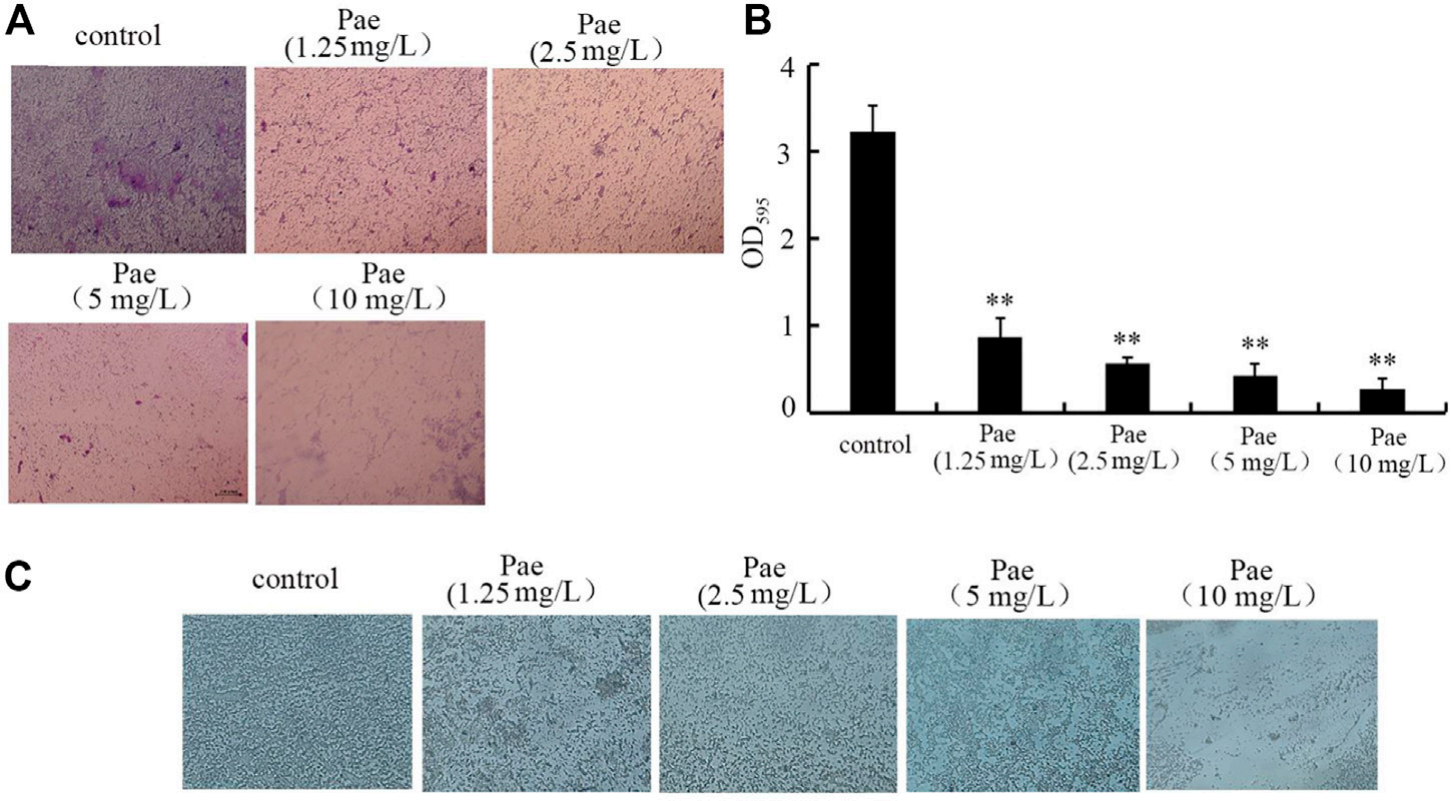
Effect of paeoniflorin treatment on biofilm formation of *P. aeruginosa* PA14. **(A)** Crystal violate staining images. **(B)** Effect of paeoniflorin treatment on amount of biofilm formation based on OD_595_ absorbance analysis. **(C)** Effect of paeoniflorin treatment on biofilm formation visualized under a light microscope. Pae, paeoniflorin. **p < 0.01 vs*.* control.

For the toxicity of *P. aeruginosa* in hosts, biofilm formation is one of the crucial virulence factors (Skariyachan et al., 2018). Based on both the crystal violate staining and analysis of OD_595_ absorbance, treatment with 1.25–10 mg/L paeoniflorin could significantly inhibit the biofilm formation of *P. aeruginosa* PA14 (Figures 6A, B). Besides these, the role of 1.25–10 mg/L paeoniflorin in suppressing biofilm formation of *P. aeruginosa* PA14 was further confirmed by the observation under the light microscopy (Figure 6C). These observations indicated the property of paeoniflorin to inhibit *P. aeruginosa* biofilm formation.

### Effect of paeoniflorin treatment on virulence genes required for the formation of *P. aeruginosa* biofilm

**FIGURE 7:**
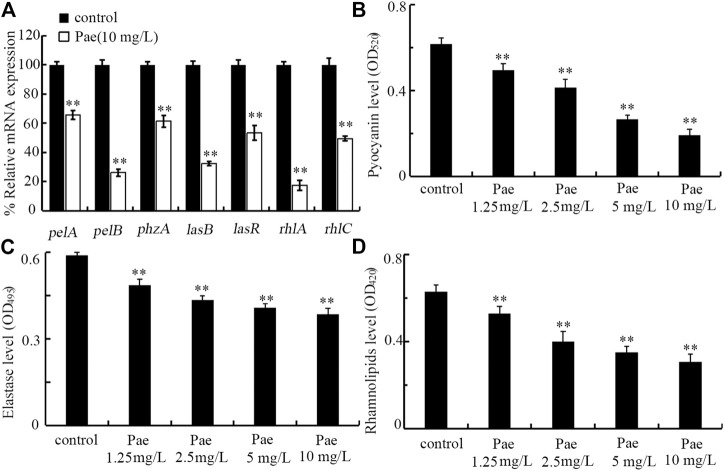
Effect of paeoniflorin treatment on expressions of virulence genes and virulence factors of pyocyanin, elastase, and rhamnolipid. **(A)** Effect of 10 mg/L paeoniflorin treatment on expressions of virulence genes required for *P. aeruginosa* biofilm formation. **(B–D)** Effect of paeoniflorin treatment on levels of pyocyanin, elastase, and rhamnolipid. Pae, paeoniflorin. **p < 0.01 vs*.* control.

In *P. aeruginosa*, the biofilm formation is controlled by some virulence genes (Phillips and Schultz, 2012; Wei and Ma, 2013). After treatment with 10 mg/L paeoniflorin, expressions of *pelA*, *pelB*, *phzA*, *lasB*, *lasR*, *rhlA*, and *rhlC* were all significantly decreased compared to control (Figure 7A). Therefore, paeoniflorin treatment could suppress expressions of virulence genes required for formation of *P. aeruginosa* biofilm.

### Effect of paeoniflorin treatment on virulence factors of pyocyanin, elastase, and rhamnolipid

In *P. aeruginosa*, pyocyanin, elastase, and rhamnolipid are important virulence factors required for biofilm formation (Zezzi do Valle Gomes and Nitschke, 2012; Das et al., 2015; Ruffin et al., 2016). After the treatment, we found that treatment with 1.25–10 mg/L paeoniflorin could significantly decrease the levels of pyocyanin, elastase, and rhamnolipid in *P. aeruginosa* PA14 (Figures 7B–D). Therefore, paeoniflorin treatment could not only inhibit expressions of virulence genes required for biofilm formation, but also suppress related virulence factors.

### Effect of paeoniflorin treatment on motility of *P. aeruginosa*

In *P. aeruginosa*, the swimming motility is a unicellular behavior requiring a functional polar flagellum (Blair, 2003). After the treatment, the swimming diameter of *P. aeruginosa* PA14 was significantly reduced by treatment with 2.5–10 mg/L paeoniflorin (Supplementary Figure S2A). Besides this, both the diameter of swarming motility and the twitching diameter of *P. aeruginosa* PA14 were also be obviously decreased by treatment with 2.5–10 mg/L paeoniflorin (Supplementary Figures S2B, C). In contrast, treatment with 1.25 mg/L paeoniflorin did not affect the swimming diameter, diameter of swarming motility, and the twitching diameter of *P. aeruginosa* PA14 (Supplementary Figures S2A–C).

## Discussion

Infection with *P. aeruginosa* is associated with some infectious diseases, including the sepsis (Cheluvappa et al., 2009; Mittal et al., 2009; Bassetti et al., 2018; Rafeq and Igneri, 2022). As one of the most feared nosocomial pathogens, treatment of *P. aeruginosa* infection is challenging due to the limited choices of antibiotics (Ibrahim et al., 2020). In this study, using lifespan as the endpoint, we observed that treatment with paeoniflorin in the range of 1.25–10 mg/L could significantly extend the lifespan of nematodes after *P. aeruginosa* PA14 infection, although 1.25–10 mg/L paeoniflorin treatment could not recover the lifespan of *P. aeruginosa* PA14 infected nematodes to the control level (Figure 1B). Especially, treatment with 10 mg/L paeoniflorin could increase 1.65 folds of mean lifespan of nematodes infected with *P. aeruginosa* PA14 (Figure 1B). In addition, treatment with 2.5–10 mg/L paeoniflorin also inhibited the ROS production and increased the locomotion behavior in nematodes after *P. aeruginosa* PA14 infection (Supplementary Figure S1A, B). In *C. elegans*, previous study has indicated the beneficial effect of paeoniflorin treatment in inhibiting Aβ proteotoxicity by regulating oxidative stress and heat shock stress responses (Ai et al., 2018). More recently, it was observed that treatment with paeoniflorin could attenuates polystyrene nanoparticle-induced reduction in reproductive capacity and increase in germline apoptosis through suppressing DNA damage checkpoints in C. elegans (Hua et al., 2023b). The observations here further indicated the potential of paeoniflorin treatment used for inhibiting *P. aeruginosa* infection in the clinical. Besides the bacterial infection, it was also found that the paeoniflorin treatment could also suppress *Candida albicans* infection by inhibiting expressions of Th1 and Th17 cells in mice (Kong et al., 2018). Therefore, paeoniflorin may have the potential in inhibiting both bacterial and fungal infections in the hosts. Treatment with 25 and 50 mg/L paeoniflorin showed the more effective effect against the toxicity of *P. aeruginosa* PA14 infection on longevity (data not shown). Given the principle of selecting the lowest possible drug dose for the intervention, we did not further examine the effect of paeoniflorin at concentrations of 25 and 50 mg/L.

In *C. elegans*, severe accumulation in intestinal lumen is one of the crucial contributors to the toxicity of both bacterial and fungal pathogen infections (Aballay and Ausubel, 2002; Sun et al., 2016). For the underlying mechanisms of the observed beneficial effect of paeoniflorin treatment against *P. aeruginosa* infection, we first found that 1.25–10 mg/L paeoniflorin treatment could obviously suppress the accumulation of *P. aeruginosa* PA14 in intestinal lumen of nematodes (Figures 2A, B). That is, treatment with paeoniflorin is helpful for nematodes to excrete the *P. aeruginosa* PA14 out of the body, which in turns helps the animals to extend their lifespan after *P. aeruginosa* infection. Xuebijing is a Traditional Chinese Medicine used for the treatment of sepsis in the clinical (Zhou et al., 2017; Li C. et al., 2021). Our recent study has demonstrated that administration with Xuebijing could help the nematodes to increase their lifespan and reduce pathogen accumulation in intestinal lumen after infection with *P. aeruginosa* PA14 (Zhang et al., 2022a). *Paeonia lactiflora* (Chishao) is one important herb medicines in Xuebijing, and the paeoniflorin accounted for 85.5% of total dose of monoterpene glycosides in Chishao (Cheng et al., 2016). Our findings suggested that the paeoniflorin may contribute to the beneficial effect of Xuebijing against *P. aeruginosa* infection and accumulation in the hosts to a certain degree.

For the mechanisms of the observed beneficial effect of paeoniflorin treatment against *P. aeruginosa* infection, we further provided the underlying molecular basis. We provided two aspects of evidence to prove the requirement of p38 MAPK, PCD-related, and Wnt signals for beneficial effect of paeoniflorin treatment against *P. aeruginosa* infection. On the one hand, the beneficial effect of paeoniflorin in increasing lifespan of *P. aeruginosa* PA14 infected nematodes could be inhibited by RNAi of *pmk-1*, *egl-1*, and *bar-1* (Figure 3A). On the other hand, the beneficial effect of paeoniflorin in reducing accumulation of *P. aeruginosa* PA14 in intestinal lumen could also be suppressed by RNAi of *pmk-1*, *egl-1*, and *bar-1* (Figure 3B). In *C. elegans*, mutation of *pmk-1*, *egl-1*, and *bar-1* caused susceptibility to toxicity of bacterial infection (Aballay and Ausubel, 2001; Troemel et al., 2006; Irazoqui et al., 2008). These observations suggested that the formation of paeoniflorin beneficial in suppressing bacterial infection required the functions of p38 MAPK, PCD-related, and Wnt signals. More importantly, our data demonstrated that p38 MAPK, PCD-related, and Wnt signals regulated the effect of paeoniflorin in inhibiting bacterial infection by affecting accumulation of pathogens in the body of hosts.

In this study, we further found that paeoniflorin could reverse the tendency of decrease in *pmk-1*, *egl-1*, and *bar-1* expressions induced by *P. aeruginosa* PA14 infection (Figure 4). This suggested that the paeoniflorin could inhibit bacterial infection in hosts by targeting specific proteins, such as the PMK-1, EGL-1, and BAR-1 in nematodes. That is, during the inhibition in bacterial infection, paeoniflorin has certain pharmacological targets in the hosts. The paeoniflorin could also affect other biological processes through acting on other pharmacological targets. For example, paeoniflorin could ameliorate colonic fibrosis by inhibiting Leptin/LepRb in rats (Tian et al., 2022).

Moreover, both time-kill assay and agar diffusion assay indicated that treatment with 1.25–10 mg/L paeoniflorin did not have obvious anti-*P. aeruginosa* activity (Figures 5A, B). These results suggested that, in *C. elegans*, the observed anti-bacterial infection property of paeoniflorin was not directly due to the possible effect of anti-bacterial activity for paeoniflorin. This also demonstrated that the observed reduction in intestinal accumulation of *P. aeruginosa* PA14 was not due to the anti-bacterial activity after paeoniflorin treatment. This further supports the important function of paeoniflorin treatment in helping animals to excrete *P. aeruginosa* PA14 out of their body.

In this study, although we did not detect the anti-bacterial infection property of paeoniflorin, we observed that treatment with 1.25–10 mg/L paeoniflorin had noticeable inhibitory effect on biofilm formation of *P. aeruginosa* PA14 (Figure 6). The biofilm of *P. aeruginosa* is built mostly by extracellular polymeric substances, and act as the scaffold to encase *P. aeruginosa* cells together on surfaces (Thi et al., 2020). *P. aeruginosa* has been considered as bacterium with the potential to produce robust biofilms, which causes severe problems in immunocompromised patients, including chronic infections and long-term persistence (Lee and Yoon, 2017). Our data implied that treatment with paeoniflorin will be useful to enhance the used antibacterial agents during treatment for *P. aeruginosa* infection in the clinical. Previous studies have shown that the paeoniflorin could inhibit the biofilm formation of some other bacteria, such as *Klebsiella pneumoniae* and *Streptococcus suis* (Qian et al., 2019; Li J. et al., 2021; Li et al., 2022). Besides the bacterial pathogen, it was also observed that the paeoniflorin treatment could inhibit the biofilm formation of *Candida albicans* (Kong et al., 2018). That is, paeoniflorin treatment may have the potential to be used to suppression biofilm formation of both bacterial and fungal pathogens.

For the observed function of paeoniflorin treatment in inhibiting biofilm formation of *P. aeruginosa*, we provided the underlying molecular basis by analyzing the expressions of related virulence genes. We found that paeoniflorin treatment could inhibited transcriptional expressions of *pelA*, *pelB*, *phzA*, *lasB*, *lasR*, *rhlA*, and *rhlC* (Figure 7A). In *P. aeruginosa*, PelA and PelB can organize a modification and secretion complex, which is essential for Pel polysaccharide-dependent biofilm formation (Marmont et al., 2017). PhzA contributed to the production of phenazine pyocyanin, which is important for *P. aeruginosa* biofilm formation (Das et al., 2015; Sun et al., 2019). *P. aeruginosa* uses quorum sensing to coordinate their biofilm formation, and *lasB* and *lasR* are quorum sensing genes (Ruffin et al., 2016). LasR is a quorum sensing transcriptional regulator, and LasB is the elastase. RhlA and RhlC are key enzymes for extracellular rhamnolipid biosynthesis, which is required for biofilm formation of *P. aeruginosa* (Rahim et al., 2001; Zezzi do Valle Gomes and Nitschke, 2012). Therefore, paeoniflorin treatment could inhibit the biofilm formation by suppressing expressions of virulence genes required for this biological process in *P. aeruginosa*.

Swimming motility is a movement depending on the flagellum (Kearns, 2010). Swimming motility is an important mechanism for bacterial pathogens to adhere to mucosal surfaces and to cause the infection (Tan et al., 2014). In this study, we observed that the motility of *P. aeruginosa* PA14, such as swimming motility, swarming motility, and twitching motility could be further suppressed by 2.5–10 mg/L paeoniflorin treatment (Supplementary Figures S2A–C). This suggested that treatment with paeoniflorin is helpful for inhibiting *P. aeruginosa* infection by suppressing its virulence factor of swimming motility. In bacteria, there is a definition of “motility-to-biofilm transition” (Guttenplan and Kearns, 2013). Our data indicated the effect of paeoniflorin in inhibiting both bacterial biofilm formation and bacterial motility, which suggested that the paeoniflorin can play its function at different developmental stages for bacterial pathogens. Effect of certain compounds in inhibiting both bacterial biofilm formation and bacterial motility has also been frequently found in other published reports (Ye et al., 2022; Zahmatkesh et al., 2022).

Besides the alterations in expressions of virulence genes, we further found that treatment with 1.25–10 mg/L paeoniflorin could significantly reduce the levels of pyocyanin, elastase, and rhamnolipid in *P. aeruginosa* PA14 (Figures 7B–D). Pyocyanin is a blue-green pigment involved in the control of ion transport, cell movement, and biofilm formation of *P. aeruginosa* (Gellatly and Hancock, 2013; Das et al., 2015). Rhamnolipids are tenso-active glycolipids containing one or two L-rhamnose molecules, and involved in the biofilm formation of *P. aeruginosa* (Boles et al., 2005). The elastase promoted the *P. aeruginosa* biofilm formation partly through the rhamnolipid-mediated regulation (Yu et al., 2014). Therefore, treatment with paeoniflorin could further affect the virulence factors involved in *P. aeruginosa* biofilm formation. More importantly, these observations further supported the detected decrease in expressions of *phzA*, *lasB*, *rhlA*, and *rhlC* (Figure 7A).

## Conclusion

Together, in this study, we used *C. elegans* as a host model to investigate potential effect of paeoniflorin treatment against *P. aeruginosa* infection. We found that paeoniflorin treatment could increase the lifespan of *P. aeruginosa* infected nematodes. After the infection, this observed beneficial effect of paeoniflorin treatment was partially due to the reduction in *P. aeruginosa* accumulation in intestinal lumen. Moreover, the observed beneficial effect of paeoniflorin treatment was also associated with inhibition in *P. aeruginosa* biofilm formation. In *C. elegans*, the beneficial effect of paeoniflorin treatment in increasing lifespan and in reducing *P. aeruginosa* accumulation after the infection was dependent of p38 MAPK, PCD-related, and Wnt signals. The beneficial effect of paeoniflorin treatment in suppressing *P. aeruginosa* biofilm formation was associated with decrease in expressions of related virulence genes and inhibition in pyocyanin, elastase, and rhamnolipid levels. Our results suggested the usefulness of paeoniflorin against *P. aeruginosa* infection and in decreasing *P. aeruginosa* virulence in the hosts.

## Data Availability

The original contributions presented in the study are included in the article/Supplementary Materials, further inquiries can be directed to the corresponding author.

## Abbreviations

ANOVA, analysis of variance; CFU, colony-forming unit; LB, Luria-Bertani; NGM, nematode growth medium; qRT-PCR, quantitative real-time polymerase chain reaction.
