# Supplementary material for: Treatment with paeoniflorin increases lifespan of Pseudomonas aeruginosa infected Caenorhabditis elegans by inhibiting bacterial accumulation in intestinal lumen and biofilm formation
Source: Front Pharmacol. 2023 Mar 27;14:1114219. doi: 10.3389/fphar.2023.1114219 (PMC10083309; doi:10.3389/fphar.2023.1114219)
Supplement: Supplementary file 1 [file DataSheet1.doc]

**Supporting Information:**

**
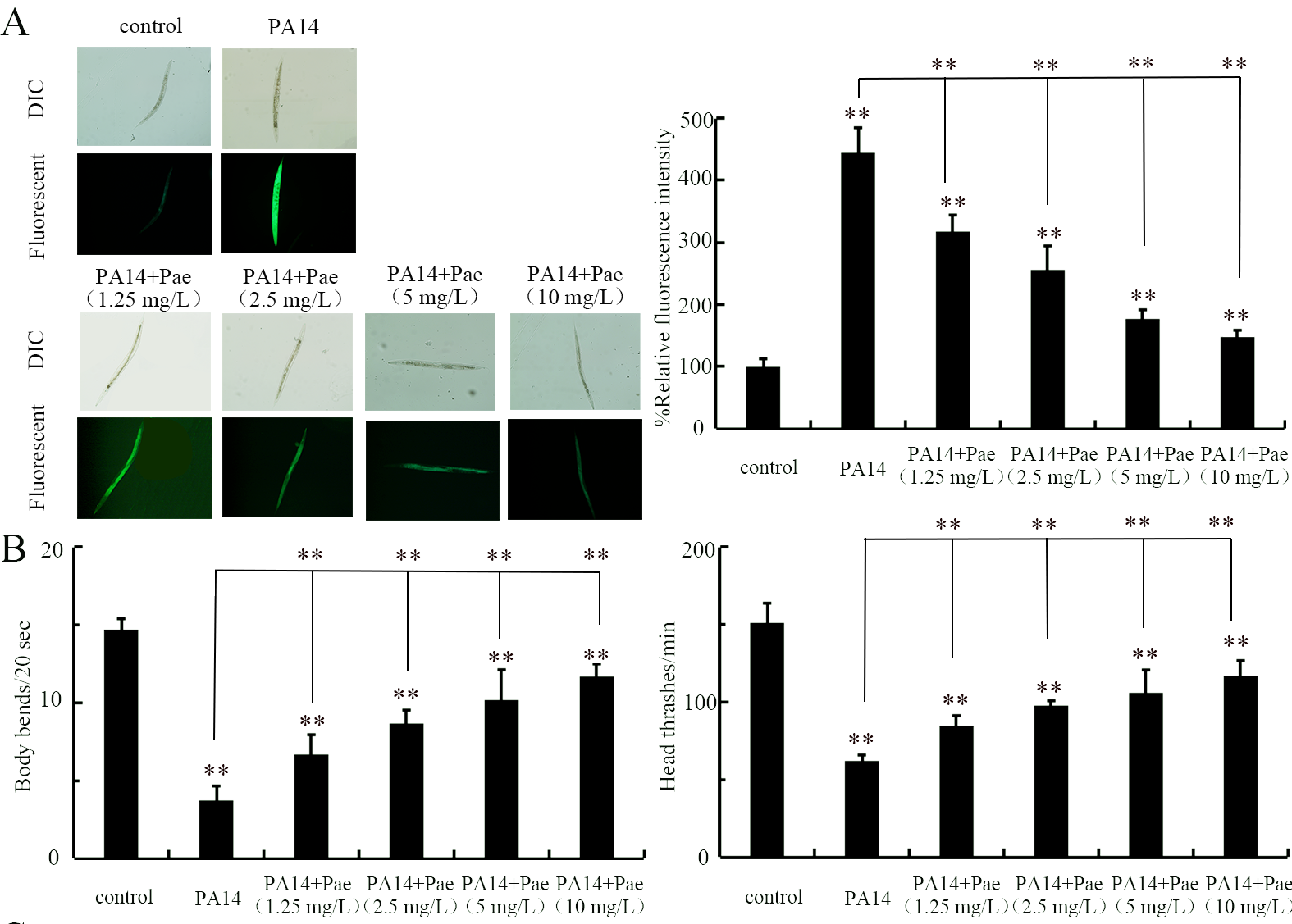
**

**Figure S1.** Effect of paeoniflorin treatment on ROS production (A) and locomotion behavior (B) in nematodes infected with *P. aeruginosa* PA14. Pae, paeoniflorin. ^**^*P* < 0.01 *vs* control (if not specially indicated).

**
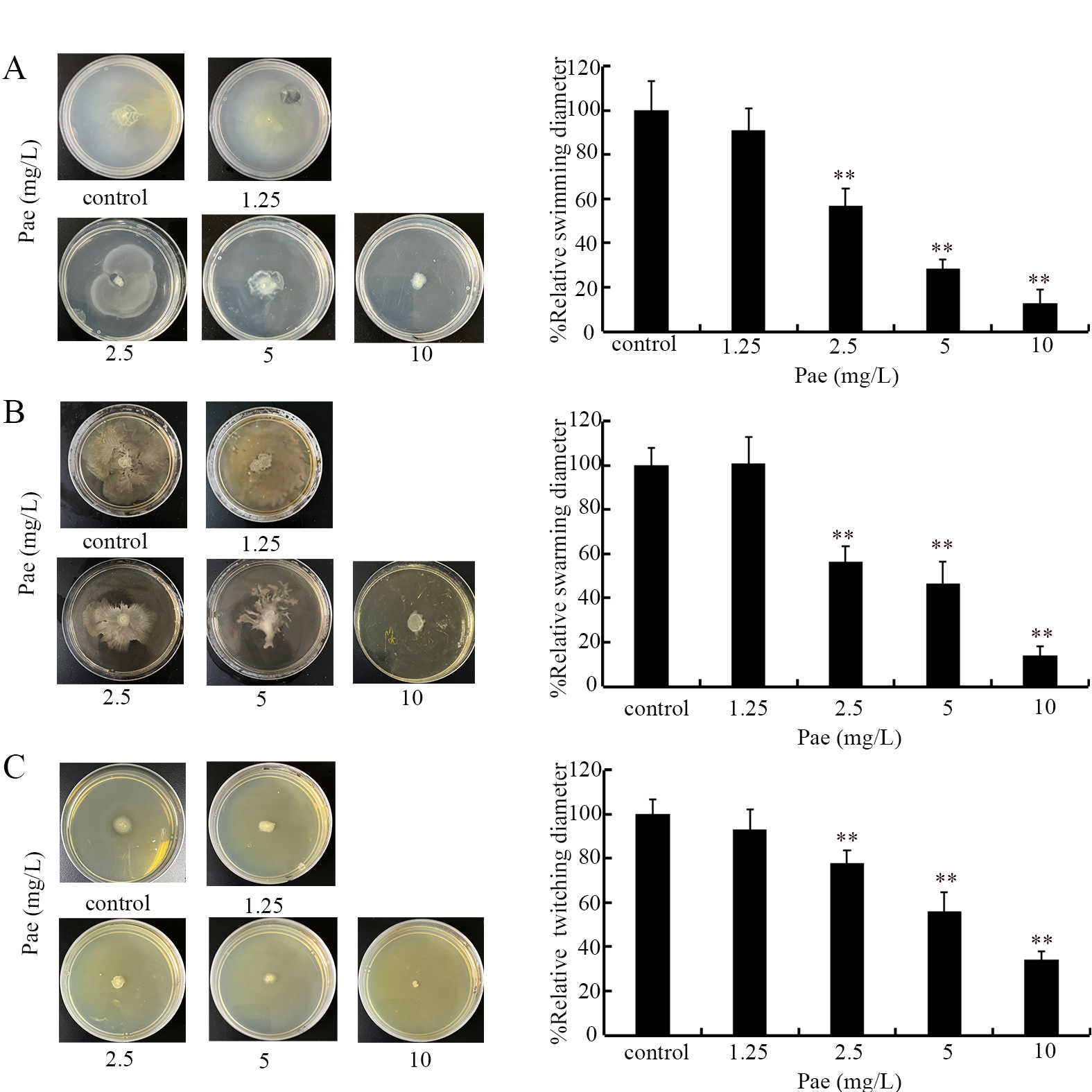
**

**Figure S2.** Effect of paeoniflorin treatment on swimming motility (A), swarming motility (B), and twitching motility (C) of *P. aeruginosa* PA14. Pae, paeoniflorin. ^**^*P* < 0.01 *vs* control.

**Table S1.** Primer information for qRT-PCR in *C. elegans*

| Gene | Forward primer (5’-3’) | Reverse primer (5’-3’) |
| --- | --- | --- |
| *pmk-1* | TCCGACTCCACGAGAAGGAT | CACGATATGTACGACGGGCA |
| *bar-1* | CCTAATTTGCACGCTACGGC | TCGGCATGATCGGAATGGAG |
| *egl-1* | GCCTCAACCTCTTCGGATCT | GCACATTGCTGCTAGCTTGG |
| *tba-1* | TCAACACTGCCATCGCCGCC | TCCAAGCGAGACCAGGCTTCAG |

**Table S2.** Primer information for qRT-PCR in PA14

| Gene | Forward primer (5’-3’) | Reverse primer (5’-3’) |
| --- | --- | --- |
| *pelA* | ATTACCTGGCTGTTCCAGGC | CCTTCGCCTATCTGTCGGTC |
| *pelB* | GGTCTCTTCCTGCAACTGCT | CAGCGTTATCCGAAACAGCG |
| *phzA* | AACGGTCAGCGGTACAGGGAAC | AACGGTCAGCGGTACAGGGAAAC |
| *lasB* | AACCGTGCGTTCTACCTGTT | CGGTCCAGTAGTAGCGGTTG |
| *lasR* | CTGTGGATGCTCAAGGACTAC | AACTGGTCTTGCCGATGG |
| *rhlA* | TGGCCGAACATTTCAACGT | GATTTCCACCTCGTCGTCCTT |
| *rhlC* | GCCATCCATCTCGACGGAC | CGCAGGCTGTATTCGGTG |
| *pvdQ* | GCCGAGGAGATCGTCACC | CAGGCGTAGAAGATGTCGGA |
